# Malignant transformation of oral leukoplakia: a retrospective cohort study of 218 Chinese patients

**DOI:** 10.1186/1471-2407-10-685

**Published:** 2010-12-16

**Authors:** Wei Liu, Yu-Feng Wang, Hai-Wei Zhou, Peng Shi, Zeng-Tong Zhou, Guo-Yao Tang

**Affiliations:** 1 Department of Oral Mucosal Diseases, Ninth People's Hospital, Shanghai Jiao Tong University School of Medicine, Shanghai Key Laboratory of Stomatology, China. No. 639 Zhizaoju Road, Shanghai 200011, China

## Abstract

**Background:**

Oral leukoplakia (OL) is the best-known potentially malignant disorder. A new binary system to grade dysplasia was proposed by WHO, but the biological significance in predicting malignant transformation risk is unknown. The objective of this study is to estimate the rate of malignant transformation in a long-term follow-up cohort, explore the usefulness of the new binary system of grading dysplasia and identify significant risk factors of OL malignant transformation in China.

**Methods:**

A total of 218 patients with clinical and histopathologic diagnosis of OL were retrospectively reviewed. They were selected among all archived files at the Department of Oral Mucosal Diseases, Ninth People's Hospital, Shanghai Jiao Tong University School of Medicine. The mean follow-up period was 5.3 years.

**Results:**

Among 218 cases, 39 (17.9%) OL patients developed oral cancer, with a mean duration of 5.2 years. Cox regression analysis revealed that dysplasia was an independent risk factor for OL malignant transformation, but age, gender, lesion site, diet habit, smoking and ethanol intake were not risk factors. High-risk dysplastic OL was associated with a 4.57-fold (95% confidence interval, 2.36-8.84; P < 0.001) increased risk of malignant transformation, compared with low-risk dysplasia. Consistent with this result, high-risk dysplastic OL had signicantly higher malignant incidence than low-risk dysplasia, particularly during the first 2-3 years of follow-up, by Kaplan-Meier analysis (Log-rank test, P < 0.001).

**Conclusions:**

The new binary system's function in predicting OL malignant transformation risk was investigated in this survey. The utilization of high-risk dysplasia as a significant indicator for evaluating malignant transformation risk in patients with OL was suggested, which may be helpful to guide treatment selection in clinical practice.

## Background

Oral squamous cell carcinoma (OSCC) is widely recognized as the most common type of head and neck cancer, with a ~50% survival rate over 5 years despite various treatments in the past three decades [1,2]. Oral leukoplakia (OL) is defined as "A white plaque of questionable risk having excluded (other) known diseases or disorders that carry no increased risk for cancer", which is the best-known potentially malignant disorder of the oral mucosa [3,4]. Reports indicate that 15.8-48.0% of OSCC patients were associated with OL when diagnosed [5-8]. Pooled estimate of annual rate of OL malignant transformation is 1.36% (95% confidence interval, 0.69%-2.03%) in various populations and geographical areas [9]. Possibly due to the rarity of cohort studies in developing nations, a concrete conclusion on the global annual rate of OL transformation is not currently available [10].

Histopathologically, oral epithelial dysplasia currently is the most important prognostic indicator for determining the malignant transformation risk of OL [11]. Traditionally, OL lesions are classified as non-dysplasia (hyperplasia) and dysplasia (mild, moderate or severe). At a workshop coordinated by the WHO Collaborating Centre for Oral Cancer and Precancer in the United Kingdom on issues related to oral potentially malignant disorders, a binary classification (no/questionable/mild-low risk; moderate/severe-high risk) of the lesion was recommended. The move was intended to reduce the subjectivity inherent in grading dysplasia, which may increase the likelihood of agreement between pathologists [12]. Kujan et al. recently tested the new binary system of grading oral dysplasia and supported this view [13]. However, the biological significance of this grading system needs to be researched in longitudinal studies to explore its relevance in predicting malignant transformation risk of epithelial precursor lesions [12].

The risk factors of clinical features and lifestyle habits associated with transformation of OL into carcinoma have been evaluated in previous studies. Napier and Speight recently reviewed clinical predictors of malignant transformation in oral leukoplakia, such as age, gender and lesion site, but the results from different study populations vary [10]. The role of smoking and ethanol intake as important risk factors related to malignant transformation remains controversial and unclear [14-16]. Thus, assessment of these factors for OL malignant transformation is still needed.

The objective of the present study is to estimate the malignant transformation rate of a retrospective cohort of 218 patients with OL (mean follow-up of 5.3 years), explore the usefulness of the new binary system of grading dysplasia and identify significant risk factors of OL malignant transformation in China.

## Methods

### Study population

All archived files of patients with the clinical and pathologic diagnosis of OL from 1978 to 2008 were retrospectively reviewed in the Department of Oral Mucosal Diseases, Ninth People's Hospital, Shanghai Jiao Tong University School of Medicine. All clinical history and follow-up data were obtained from the archived files. Information regarding gender, age, site of lesions at the time of the initial diagnosis of OL was all documented in detail. Diet habit, history of smoking and ethanol intake were also collated through the files. According to the definition of OL, "A white plaque of questionable risk having excluded (other) known diseases or disorders that carry no increased risk for cancer", the exclusion criteria were as follows:

I. Any patient without the initial histopathologic examination of OL and development of OSCC during a follow-up period by biopsy or surgery.

II. Any patient with the clinical history and histopathologic changes of oral white or predominantly white oral benign diseases, for example, linea alba, leukoedema, leukokeratosis; and oral precancerous conditions such as discoid lupus erythematosus and lichen planus.

III. Any patient with diagnosis of OL concomitant OSCC at the first visit.

IV. Any patient with a follow-up period of less than 12 months.

Based on these criteria, 218 patients with OL were selected to be retrospectively reviewed in the cohort. This study was approved by the institutional review board of Ninth People's Hospital, Shanghai Jiao Tong University School of Medicine.

### Histopathologic examination

The histopathologic diagnosis of all cases was determined by an oral pathologist on duty from the Department of Oral Pathology, Ninth People'sHospital, Shanghai Jiao Tong University School of Medicine. The confirmation diagnosis and reclassification was performed by another oral pathologist (J. L.). The WHO criteria for OL were used when examining the histopathology of the sections [17]. According to the binary grading system newly proposed by WHO [12], reexamination of the sample confirmed the diagnosis of epithelial dysplasia. The architecture (a total of 7 scoring) and cytology (a total of 9 scoring) criteria for epithelial dysplasia were as follows:

Architecture: 1) Irregular epithelial stratification; 2) Loss of polarity of basal cells; 3) Drop-shaped rete ridges; 4) Increased number of mitotic figures; 5) Abnormally superficial mitoses; 6) Premature keratinization in single cells; 7) Keratin pearls within rete ridges.

Cytology: 1) Abnormal variation in nuclear size; 2) Abnormal variation in nuclear shape; 3) Abnormal variation in cell size; 4) Abnormal variation in cell shape; 5) Increased nuclear-cytoplasmic ratio; 6) Increased nuclear size; 7) Atypical mitotic figures; 8) Increased number and size of nucleoli; 9) Hyperchromasia.

We reclassified all lesions as low-risk dysplasia and high-risk dysplasia in the present study. A low-risk lesion was based on observing less than four architectural changes or less than five cytological changes. A high-risk lesion was based on observing at least four architectural changes and five cytological changes.

### Statistical analysis

A descriptive analysis was performed on clinical and pathologic factors. The χ^2 ^test and Fisher exact test were employed to assess the association among categorical variables. For time-to-event analysis, Kaplan-Meier curves were plotted and log-rank test was used. Cox proportional hazards models were utilized for clinicopathological factors in prediction of malignant transformation risk. Hazard ratios (HR) with 95% confidence interval (95% CI) and P values were reported. All tests were two-sided, and P values < 0.05 were considered statistically significant.

## Results

### Patient Characteristics

A total of 218 patients were enrolled in this retrospective study, with a mean follow-up period of 5.3 years. Of these, 39 (17.9%) patients developed invasive oral cancer, with the mean time of malignant transformation of 5.2 years.

The baseline characteristics of OL are presented in Table 1. For all the subjects, the gender ratio was equal (110 males: 108 females). The average age at diagnosis was 52.7 years old (range 21-84). The peak incidence was fifth decade of life (33.0%). Tongue was affected in 51.4% patients with OL, followed by buccal mucosa (32.6%). Few lesions were located on the floor of mouth and lip. There were 12.8% patients with spicy dietary habit. The history of smoking and ethanol intake were observed in 29.8% and 6.9% cases, respectively. We found 180 (82.6%) OL cases were low-risk dysplastic lesions and 38 (17.4%) OL cases were high-risk dysplastic lesions. Table 2 illustrates the association between OL and clinical parameters. Differences in age, gender, lesion site, diet habit, smoking, and ethanol intake were not observed between the untransformed and transformed OL.

**Table 1:** Baseline characteristics of oral leukoplakia

	Patients
Characteristic	n (%)
Total	218
Age, y	
Mean (SD)	52.7 (11.2)
Range	21-84
< 40	24 (11.0)
40 - 49	59 (27.1)
50 - 59	72 (33.0)
>= 60	63 (28.9)
Gender	
Female	108 (49.5)
Male	110 (50.5)
Site	
Tongue	112 (51.4)
Buccal mucosa	71 (32.6)
Gingiva	14 (6.5)
Palate	13 (6.0)
Floor of mouth	7 (3.2)
Lip	1 (0.5)
Diet habit	
Bland	177 (81.2)
Spicy	28 (12.8)
Missing	13 (6.0)
Smoking	
Never	145 (66.5)
Past and present	65 (29.8)
Missing	8 (3.7)
Ethanol intake	
Never	193 (88.5)
Past and present	15 (6.9)
Missing	10 (4.6)
Epithelial dysplasia	
Low-risk	180 (82.6)
High-risk	38 (17.4)

**Table 2:** Characteristics of untransformed (UT) and malignant transformed (MT) oral leukoplakia

	Oral leukoplakia, n (%)		
		
Characteristic	UT	MT	P-value
	n = 179	n = 39	
Age, y			0.846
< 60	128 (71.5)	27 (69.2)	
>= 60	51 (28.5)	12 (30.8)	
Gender			0.113
Female	84 (46.9)	24 (61.5)	
Male	95 (53.1)	15 (38.5)	
Site			0.051
Tongue	86 (48.0)	26 (66.7)	
Others sites	93 (52.0)	13 (33.3)	
Diet habit			0.179
Bland	143 (84.6)	34 (94.4)	
Spicy	26 (15.4)	2 (5.6)	
Missing	10 (-)	3 (-)	
Smoking			0.176
Never	115 (66.9)	30 (78.9)	
Past and present	57 (33.1)	8 (21.1)	
Missing	7 (-)	1 (-)	
Ethanol intake			0.484
Never	159 (93.5)	34 (89.5)	
Past and present	11 (6.5)	4 (10.5)	
Missing	9 (-)	1 (-)	

### Oral cancer-free survival (OCFS) analysis of risk factors

**Figure 1:**
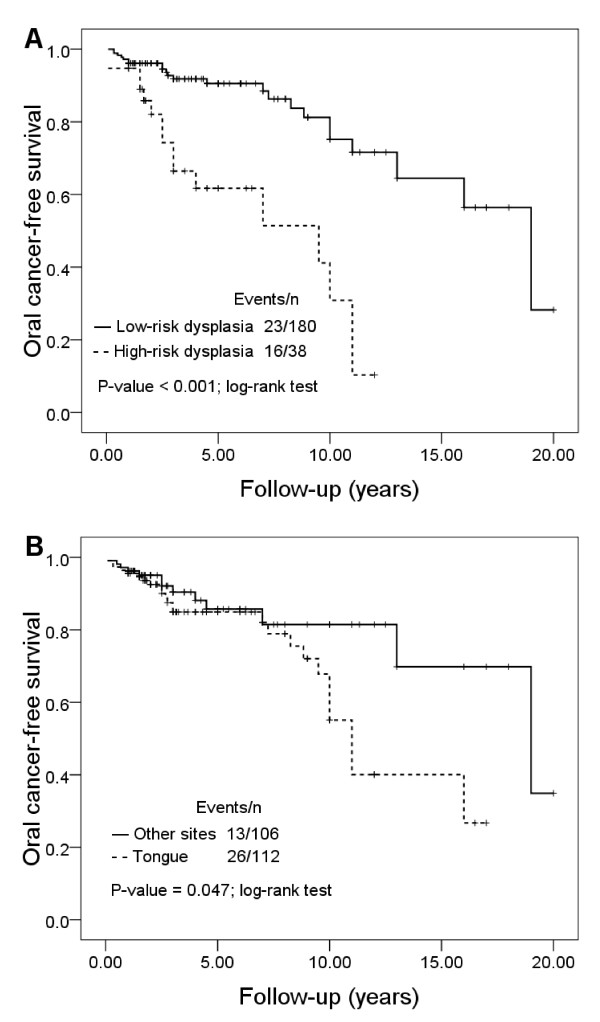
**Oral cancer-free survivals (OCFS)**. (**A**) by low-risk and high-risk dysplasia. (**B**) by tongue and other sites of lesion.

To investigate the time to malignant event of OL, we performed the Kaplan-Meier analysis for cancer-free survival by clinicopathological parameters. Dysplasia and lesion site were significant parameters by Log-rank test (Figure 1). Among 218 OL lesions, 23 of 180 low-risk dysplasia and 16 of 38 high-risk dysplasia developed into cancer, respectively. We observed that a high-risk dysplastic OL was associated with an increased oral cancer risk, particularly during the first 2-3 years of the follow-up (Log-rank test, P < 0.001; Figure 1A). The 5-year OCFS rate of low-risk dysplasia was 90.6% (95%CI, 0.86-0.96) compared with 61.7% (95%CI, 0.44-0.80) for high-risk dysplasia. The Kaplan-Meier curve also depicts another high incidences of malignant events for patients with dysplasia occured during the 8-10 years of follow-up. In addition, OL located at tongue had higher malignant incidence than those found at other sites, particularly after about 10 years of follow-up (Log-rank test, P = 0.047; Figure 1B).

### Cox regression analysis of risk factors for OL malignant transformation

An analysis of risk factors of the transformation of OL into cancer was performed by the cox proportional hazards model (Table 3). In the cox regression analysis, age, gender, lesion site, diet habit, smoking and ethanol intake were not found to be significantly associated with the malignant development. The risk of malignant transformation of OL located at tongue may be higher than at other sites (HR = 1.97, 95% CI, 0.99-3.93; P = 0.053). Importantly, the degree of dysplasia was an independent risk factor associated with malignant transformation. The high-risk dysplastic lesions were associated with 4.57-fold (95% CI, 2.36-8.84; P < 0.001) increased the risk of malignant transformation, when compared to the low-risk dysplastic lesions.

**Table 3:** Cox regression analysis of risk factors for oral leukoplakia transformation

Characteristic	Hazard Ratio (95%CI)	P-value
Age, y		
< 60	1.00	
>= 60	1.87 (0.92-3.80)	0.086
Gender		
Female	1.00	
Male	0.56 (0.29-1.08)	0.084
Site		
Other sites	1.00	
Tongue	1.97 (0.99-3.93)	0.053
Diet habit		
Bland	1.00	
Spicy	0.41 (0.10-1.70)	0.217
Smoking		
Never	1.00	
Past and present	0.60 (0.26-1.36)	0.222
Ethanol intake		
Never	1.00	
Past and present	1.19 (0.42-3.37)	0.075
Epithelial dysplasia		
Low-risk	1.00	
High-risk	4.57 (2.36-8.84)	< 0.001

## Discussion

In the present study, we evaluated the malignant transformation rate of Chinese patients with OL, and identified the risk factors of malignant transformation during a relatively long follow-up period. Of the 218 cases, 39 (17.9%) patients developed invasive cancer, with a mean malignant transformation period of 5.2 years. Dysplasia was an independent risk factor for OL malignant transformation, but age, gender, site, diet habit, smoking and ethanol intake were not risk factors (Table 3).

In our series, according to the WHO criteria, we considered the time elapsed from the initial diagnosis of OL to the development of cancer. In this context, we excluded patients with diagnosis of OL concomitant OSCC at the first visit or patients with a followed-up period of less than 12 months after the initial diagnosis of OL. Our recorded annual malignant rate of 3.38% is higher than the rate documented in the literature (0.69%-2.03%) [9-11]. Herein, various treatments on the OL patients were not considered because few prevention studies have shown effectiveness in preventing the transformation of OL to cancer [18].

Although it is a well-known fact that the histological classification of OL lesions is imperfect, which may involve subjectivity, we cannot do without it to date [12]. Notwithstanding it had been elucidated that oral lesions with epithelial dysplasia more often develop into cancer than those with hyperplasia in previous findings [11,14,19,20], few studies have examined the risk of malignant development in different grades of dysplastic OL. In an American population, moderate or severe dysplastic OL was associated with 2.30-fold increased risk of malignant transformation, compared with mild dysplasia or hyperplasia [21]. In a Dutch study [22], OL diagnosed with moderate or severe epithelial dysplasia had a significantly higher risk of malignant development than leukoplakia with lower dysplasia grades (P < 0.01). These findings were similar to ours. The degree of dysplasia was associated with OL malignant transformation (Table 3).

According to the new binary grading system proposed by WHO, we found the high-risk dysplastic OL was associated with a 4.57-fold (P < 0.001) increased risk of malignant transformation, when compared to low-risk dysplasia by Cox regression. Consistent with this result, patients with high-risk dysplastic OL had significantly higher oral cancer incidence than low-risk dysplasia, particularly during the first 2-3 years of follow-up by Kaplan-Meier curve (P < 0.001; Figue 1A). Similar findings were reported by Ho et al and Silverman et al[14,16]. It is suggested that rigorous follow-up in the first 2-3 years for patients with diagnosis of dysplastic OL may be important to detect early malignant events.

We observed the average age at diagnosis of OL is 52.7 years, while other study populations had an average age closer to 60 years [16,20]. The peak incidence of OL was in the fifth decade of life in our study, earlier than the sixth decade in other reports [20,23]. Gender predilection is not of existence in our area, but significant gender differences (ratio M: F = 10.6: 1) were found in Taiwan, China [24]. The predominant sites of lesions are the tongue and buccal mucosa, and few lesions were located on the floor of mouth, whereas the tongue and floor of mouth were reported as the most common sites in Western countries [6,16,20]. These were probably due to the ethnic population and geographic difference in our cohorts compared to previous reports. Nevertheless, these factors were not related to malignant transformation of OL in our series. Moreover, It may be generally accepted that smoking and ethanol intake play significant roles in the development of OL, but the roles of those in the malignant transformation of OL is conflicting and yet unclear. The studies by Silverman et al [16] and Schepman et al [20] demonstrated an increased risk of malignant transformation in the non-smoking cohort, while the study by Ho et al [14] and our present study found smoking was not a significant factor in transformation risk. Ethanol intake was also not a risk factor for malignant transformation of OL [14,15]. Further prospective cohort studies are needed to investigate the roles of lifestyle habits in the malignant process of OL.

## Conclusions

In summary, we evaluated the usefulness of the new binary system of grading dysplasia proposed by WHO in prediction of OL malignant transformation risk. High-risk dysplasia was a significant indicator for OL malignant transformation. It is thus import to detect early malignant events of OL with the diagnosis of high-risk dysplasia in rigorous followed-up in the first 2-3 years.

## Competing interests

The authors declare that they have no competing interests.

## Authors' contributions

WL analyzed data and drafted the manuscript. YFW and PS collected and assembled data. HWZ participated in conception and design of the study. GYT and ZTZ coordinated and conceived the study. All authors read and approved the final manuscript.

## Pre-publication history

The pre-publication history for this paper can be accessed here:

http://www.biomedcentral.com/1471-2407/10/685/prepub
